# One-Dimensional Modeling Studies of the Gaseous Electronics Conference RF Reference Cell

**DOI:** 10.6028/jres.100.035

**Published:** 1995

**Authors:** T. R. Govindan, M. Meyyappan

**Affiliations:** Scientific Research Associates, Inc., P.O. Box 1058, Glastonbury, CT 06033-6058

**Keywords:** fluid model, hybrid model, one-dimensional, plasma processing, reference cell, rf discharge

## Abstract

A review of the one-dimensional modeling studies in the literature of the Gaseous Electronics Conference (GEC) reference plasma reactor is presented. Most of the studies are based on the fluid model description of the discharge and some utilize hybrid fluid-kinetic schemes. Both models are discussed here briefly. The models provide a basic understanding of the discharge mechanisms and reproduce several critical discharge features observed experimentally.

## 1. Introduction

Low temperature, weakly-ionized, radio-frequency (rf) discharges are commonly used in semiconductor processing. RF plasma enhanced chemical vapor deposition (PECVD) and reactive ion etching (RIE) are two key steps in integrated circuit manufacturing. Though the semiconductor industry has been recently experimenting with inductive coupled plasma (ICP) and electron cyclotron resonance (ECR) sources for high rate and large area processing, rf plasma reactors continue to be used in large-scale manufacturing. Regardless of the nature of the power source, all discharges used in processing are notoriously complex and characterized by several interacting phenomena: plasma generation of reactive species, power deposition, sheath behavior and ion acceleration, plasma-surface interaction, gas flow, and heat transfer. These phenomena seriously affect equipment and process design efforts. Indeed, it is a well-known fact that seemingly identical commercial reactors behave differently under “identical” operating conditions. This long-standing problem with plasma processing reactors has prompted researchers, under the auspices of the Gaseous Electronics Conference (GEC), to design a standard reactor as an experimental platform [1]. This GEC reactor has been used in many laboratories in the past 5 years and experimental data from different research groups are routinely compared to enhance our understanding of rf plasma processing. The preceding articles in this Special Issue describe results from experimental studies and comparison of data taken from different GEC reactors. Another aim of the GEC reference reactor related research is to provide diagnostics data for validation of discharge models. Modeling of plasma processes has been intensively pursued in the past decade to aid in the interpretation of experiments and provide an understanding of discharge mechanisms. This article reviews one-dimensional modeling studies pertinent to the GEC reference reactor in the published literature. The number of articles on the modeling of GEC reactors is rather limited and this review is based on the following studies: Sommerer and Kushner [2], Lymberopoulos and Economou [3], Meyyappan and Govindan [4], Young and Wu [5], and Riley et al. [6]. In addition, the discussion is augmented with unpublished results from our work. For a detailed general review of the discharge physics and chemistry modeling, the reader is referred to Ref. [7].

## 2. A Review of Models

Direct solution of the Boltzmann equation for rf processing problems is difficult and not commonly done, even though the Boltzmann equation is the basis for all theoretical descriptions of the plasma. Numerical modeling of discharges falls into one of two major categories: fluid and kinetic models. Fluid models consist of either two or three moments of the Boltzmann equation, along with Poisson’s equation and provide information on the number density, momentum, and energy of each charged species. Fluid models do not solve for the electron energy distribution function (EEDF); rather, they require EEDF and rate constants as input to the model. In contrast, kinetic models self-consistently solve for the EEDF and provide all of the plasma characteristics. In the following two sections, we provide a brief review of the two schemes as practiced in the studies referenced here [2–6].

### 2.1 Kinetic Schemes

The most common kinetic scheme involves integration of the Boltzmann equation with the aid of Monte-Carlo techniques to account for collisions. For a review of particle-in-cell (PIC) techniques, the reader is referred to Ref. [8] and references therein. In PIC and other statistical schemes, a large number of superparticles are needed to ensure meaningful statistics and obtain smooth results. For electronegative discharges, where ion recombination reactions are significant, even a larger number of superparticles would be required. Hence, the kinetic schemes are computationally intensive. In contrast, fluid models require much less computer time and are more robust. Kushner and coworkers [2,9] introduced a hybrid scheme which retains the advantages of both the fluid and PIC approaches and reduces the computational burden. Their approach is briefly discussed below and their results for the GEC reference reactor are discussed in Sec. 3.1.

The hybrid scheme of Kushner and coworkers [2,9] consists of three modules, namely, an electron Monte-Carlo simulator (EMCS), neutral chemistry and transport model (NCTM), and self-consistent fluid model (SCFM). The computer simulation begins with an initial guess for electron density and electric field as a function of position and phase. The EMCS is run first using 100–500 superparticles for 10–200 rf cycles. In EMCS, the trajectories of individual electron particles are tracked in time with prescribed time steps. When the number of superparticles changes due to inelastic processes such as ionization and attachment, simulated electrons are removed or added to maintain approximately the same number of simulated particles. The EED is formed by periodically recording the phase space location of each electron in the ensemble. The output of EMCS is the EEDF, electron impact rate coefficients, source terms for electrons and ions, and transport data. NCTM is then used to obtain steady-state densities of neutrals, and source and sink rates for charged species. The NCTM consists of continuity equations for each neutral species expressed in terms of a finite difference formulation. The information from both EMCS and NCTM are next used in the self-consistent fluid model. The SCFM consists of continuity equations for each charged species and Poisson’s equation. The continuity equation itself comprises of drift and diffusion mechanisms for transport and source and sink terms. The equations expressed in a finite difference form are solved using an Euler method to yield the charged species density and electric field at every call to the SCFM. The SCFM is run for 5–20 rf cycles at every call. The overall process between the three modules is iterated 100–1000 rf cycles as warranted by the timescales of the problem until a periodic steady solution is reached.

Riley et al. [6] also used a modified hybrid model in which the kinetic portion of the scheme involves direct solution of the Boltzmann equation for the electrons in one configuration-space and two velocity-space dimensions. A method of characteristics combined with a finite-difference expression for the collision integrals was used to solve the Boltzmann equation. The solution for the electrons alternated between the kinetic equations and a fluid or fluid-like “average” description. The neutral and heavy charged particles were treated with fluid equations at all times. The solution was run until convergence. Details are contained in [6].

### 2.2 Fluid Models

#### 2.2.1 Governing Equations

A detailed discussion on the derivation of moment equations, closure relations, boundary conditions and other related subjects can be found in Ref. [7]. Here, only a brief summary is given to indicate the general formulation as employed in Refs. [3–5]. The fluid model consists of mass, momentum, and energy conservation equations for each charged species *j*.

$$\frac{\partial n_{j}}{\partial t}+\nabla \cdot n_{j}v_{j}=\sum_{i}R_{ij}$$ (1)

$$\frac{\partial }{\partial t}(n_{j}m_{j}v_{j})+\nabla \cdot (n_{j}m_{j}v_{j}v_{j})=-\nabla P_{j}+q_{j}n_{j}E-n_{j}m_{j}v_{j}v_{j}.$$ (2)

$$\frac{\partial }{\partial t}w_{j}+\nabla \cdot v_{j}w_{j}=q_{j}n_{j}v_{j}\cdot E-\nabla \cdot P_{j}v_{j}+\nabla \cdot K_{j}\nabla T_{j}-\sum_{i}R_{ij}H_{i}.$$ (3)

Here *n* is the number density, *m* is particle mass, and ***v*** is average or directed velocity. The total energy comprises of kinetic energy associated with directed motion and thermal energy; $w=n\left(\frac{1}{2}mv^{2}+U\right)$. In the above equations, the sign of *q_j_* must be used: + for positive ions and − for electrons and negative ions. Other notations are as follows: *t* is time, *R_ij_* is the rate of generation or consumption of species *j* through inelastic collision *i*; *P* is species partial pressure defined as *nkT* where *k* is the Boltzmann constant and *T* is temperature; ***E*** is electric field, *v* is elastic collision frequency; *K* is thermal conductivity; and *H_i_* is energy loss following inelastic collision process *i*. In addition to Eqs. (1)–(3), we need Poisson’s equation:

$$\nabla^{2}\psi =-\frac{\rho }{\epsilon_{0}}.$$ (4)

Here *ρ* = Σ*_j_ q_j_ n_j_* is the net charge density and ***E*** = −∇*ψ* where *ψ* is potential.

The terms in the momentum equation [Eq. (2)] respectively represent time rate of change of momentum, convective acceleration or inertia, pressure gradient, particle drift due to electric field and finally, momentum loss (or drag) due to electron/gas, ion/gas elastic collisions. The momentum loss or friction term in Eq. (2) is written with the aid of an effective momentum transfer frequency *v_m_*. Equivalently, an effective momentum relaxation time *τ_m_* may be defined and the friction term is written as *nmv/τ_m_* [5]. The terms in Eq. (3) respectively represent time rate of change of total energy, convective transport of energy, energy gain from the electric field, rate of work done by pressure forces, energy transport by conduction, and energy loss due to inelastic collisions. The energy loss term in Eq. (3) includes loss from ionization, excitation, and other inelastic collisions. Alternatively an energy relaxation term may be defined as in Ref. [5] and the energy loss term then is written as *n*(*w–w*_0_)/*τ*_ϵ_ where *w*_0_ is the energy of the background gas. The heavy ions exchange energy with the background gas efficiently and their temperature is close to that of the gas. For this reason, rf discharge models commonly do not include an energy equation for ions.

The studies in Refs. [4] and [5] use the above three moment equations. In contrast, the study in Ref. [3] approximates the momentum equation by neglecting the time derivative and inertia terms in Eq. (2) and uses a drift-diffusion relation:

J=nv=sign(q)μnE−D∇n (5)

which is substituted in the continuity Eq. (1). Consistently, the kinetic energy 1/2*mv*^2^ is taken to be small compared to thermal energy *U* in Eq. (3). In Eq. (5), *μ* is the mobility and *D* is the diffusivity. The set of Eqs. (1) and (3) with flux relation Eq. (5) form the two moment approach, which has one less partial differential equation (per species) in each direction than the three moment approach and therefore results in considerable savings in computer time. While a two moment approach may be suitable to describe electron transport for reasonably high pressures, inertial effects are significant in ion transport. Recognizing this, Lymberopoulos and Economou [3] used an effective electric field $\bar{E}$ in the flux relation [Eq. (5)] for ions where $\bar{E}$ is given by

$$\frac{\partial \bar{E}}{\partial t}=\frac{q}{\mu_{i}m_{i}}\left(E-\bar{E}\right)$$ (6)

Here subscript i denotes ions. Equation (6) has been derived assuming that ion transport can be modeled as an inertially accelerating particle:

$$\frac{\partial v_{i}}{\partial t}=\frac{F}{m_{i}}=\frac{qE}{m_{i}}-\frac{qv_{i}}{\mu_{i}m_{i}}$$ (7)

The net velocity ***v***_i_ is given by $\mu_{i}\bar{E}$, and substituting this in Eq. (7) provides Eq. (6). Thus, the use of Eq. (6) attempts to consider ion inertia while preserving the simple form of Eq. (5).

#### 2.2.2 Rate Constants and Transport Data

The fluid model equations presented in the previous section need to be augmented with information on rate expressions for various species generation/loss processes and transport parameters. In the kinetic schemes described in Sec. 1, since the EEDF is a model output, information on rate processes and transport parameters were generated self-consistently using available cross-section data as input. This is not the case with fluid models. The form of EEDF has to be assumed and then the rate constant can be generated using known cross-sections from:

$$k_{j}=\int_{0}^{\infty }f(\epsilon )\sigma_{j}(\epsilon )u(\epsilon )d\epsilon .$$ (8)

Here ***k****_j_* is the rate constant for process *j*, **σ** is collision cross-section, ***ϵ*** is electron energy and *u* is electron velocity, ***ϵ*** = 1/2 *mu*^2^. It is easy to see that the utility of any fluid model critically depends on the input for rate processes, i.e., the form of EEDF assumed. The common sources for such input are dc Monte-Carlo simulations, zero-dimensional (spatially homogeneous) solution to the Boltzmann equation, and swarm experiments. Young and Wu [5] used a dc Monte-Carlo procedure which can be thought of as a “numerical swarm experiment.” This involves following a swarm of test electrons in a specified constant electric field with collisional processes modeled statistically in a manner similar to the kinetic scheme in Sec. 2.1. Young and Wu generated inelastic rate constants, mobility and diffusivity as a function of electric field-to-gas density ratio (*E*/*N*). Alternatively Lymberopoulos and Economou [3] solved the time- and space-independent Boltzmann equation for a given *E*/*N* to obtain the EEDF and computed rate constants using Eq. (8). Meyyappan and Govindan [4] used the functional relations of rate constants vs mean energy from Ref. [3].

#### 2.2.3 Boundary Conditions

To complete the fluid model description, we need statements of boundary conditions for solution variables. In one-dimensional rf discharge models, the conditions discussed below are applied to both electrodes. For electrons, the net flux at the electrode is given by the sum of two contributions: electrons lost due to recombination and electrons generated by secondary electron emission. It is usually assumed that all electrons striking the electrode are absorbed, i.e., unity sticky coefficient or zero reflection coefficient. The flux of electrons to the electrode is given by *nv*_t_/4 where *v*_t_ is the thermal velocity given by (8*kT*_e_/π*m*)^1/2^. Sometimes this flux is written *k_r_n* where *k* is a recombination coefficient and comparison with the previous expression gives *k*_r_=*v*_t_/4 [3]. Including secondary electron emission, the boundary condition for electrons becomes

$$j_{e}=\frac{n_{e}v_{t}}{4}-\gamma j_{+}.$$ (9)

Here *γ* is secondary electron emission coefficient and *j*=*nv* is the flux. In the two moment approach, flux relation [Eq. (5)] is used for *j*. Alternatively, if secondary electron emission is negligible and for a unity sticky coefficient, electron density at the electrode is assumed to be zero [4, 5]. For positive ions, the flux at the electrode is dominantly due to drift and therefore,

$$\partial n_{+}/\partial x=0.$$ (10)

The negative ions in rf discharges are repelled from the electrodes at 13.56 MHz and therefore negative ion density at the wall may be set to zero. The boundary condition for electron mean energy may take the form of an energy balance at the electrode. If the secondary electrons are assumed to emerge with an energy ***ϵ***_s_, then the energy balance is written as

$$q_{e}=\frac{nv_{t}U}{4}-\gamma j_{+}\epsilon_{s}$$ (11)

where

$$q_{e}=j_{e}U-K\frac{\partial T}{\partial x}.$$ (12)

When *γ* is zero, the electron temperature gradient is zero at the electrode. A constant electron temperature of 0.5 eV at the electrode has been assumed in all of the previous works reviewed here [3–5]. For the three moment approach, additional conditions are required in the numerical solution procedure to conveniently close the discretized set of governing equations. For this purpose, second order extrapolation conditions (zero second derivatives) for the electron and ion velocities are normally used. Finally, for Poisson’s equation, a zero-voltage condition is imposed on the grounded electrode. A sinusoidal voltage waveform is normally used at the powered electrode.

## 3. Modeling Results

It is noted that the present review is confined to one-dimensional simulations of the GEC cell and hence the only relevant dimension for modeling is the electrode gap, which is 2.54 cm. None of the one-dimensional models to date [2–6] account for the asymmetry of the discharge. Nevertheless, these studies have provided valuable information on the rf discharge mechanisms. In our present work, we have attempted to account for the asymmetry using an “effective area” approach; these results will be discussed in Sec. 3.2.

### 3.1 Hybrid Model Results

**Fig. 1:**
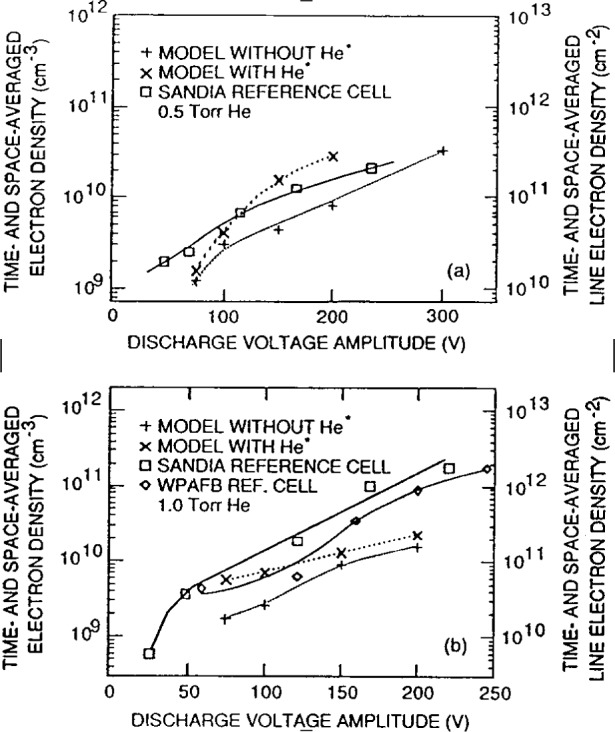
Comparison of hybrid model results and microwave interferometry measurements of electron density in helium discharges. Reproduced with permission from Ref. [2].

**Fig. 2:**
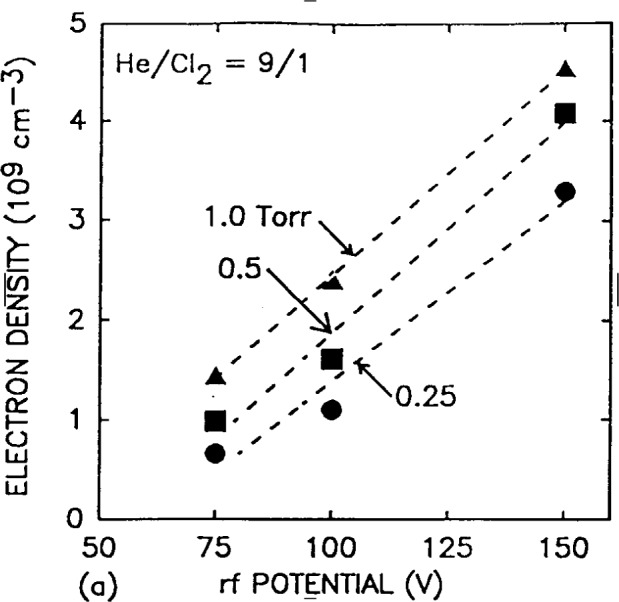
Electron and ion densities in a He/Cl_2_ discharge as predicted by hybrid model. Reproduced with permission from Ref. [9].

Kushner and coworkers [2,9] modeled a number of gas discharges corresponding to GEC cell conditions: He, N_2_, He/N_2_/O_2_ mixture, He/Cl_2_, He/HCl, He/CF_4_/O_2_ mixture and SiH_4_/NH_3_ mixture. Unfortunately, comparison with measurements was limited since experimental results for these discharges were not available at the time of their work. They provided a comparison of their modeling results with microwave interferometry measurements of line integrated density in He from two GEC reference reactors. This comparison is reproduced in Fig. 1. The simulation results in Fig. 1 are for two cases, with and without inclusion of the metastables in the model. The electron density is seen to increase by a factor of 2 to 3 when contributions from Penning reactions and multistep ionization processes are included in the model. To compare against the microwave interferometry measurements, the plasma was assumed to have no radial variation and the one-dimensional results were integrated over the 10.16 cm diameter of the electrode. This also assumes that the plasma does not spread beyond the cylinder defined by the 10.16 cm electrodes. Despite these assumptions, the results from the 1-D hybrid model are in reasonable agreement with measurements, as shown in Fig. 1. The authors believe that at high voltages, corresponding to a power density of above 10 mW/cm^2^, secondary electron emission may become important and must be included in the model in order to reproduce experimental observations. Time and spatially averaged electron density and positive ion density from a hybrid model of a 0.9/0.1 He/Cl_2_ discharge are shown in Fig. 2. The plasma density exhibits a linear dependence on rf voltage. ${\text{Cl}}_{2}^{+}$ is the dominant positive ion even though the mole fraction is 0.1. The production of He^+^ is small and in addition, charge exchange with Cl_2_ depletes the He^+^ ions rapidly.

**Fig. 3:**
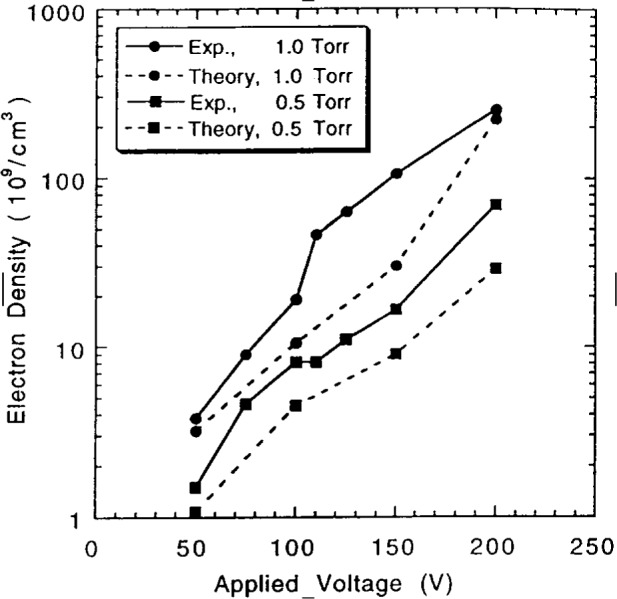
Comparison of theory and measurement of electron density in a helium discharge. Reproduced with permission from Ref. [6].

Riley et al. compared their simulation results for helium discharges with microwave interferometry measurements reported in Ref. [10] on a symmetrically (“push-pull”) driven GEC cell. This comparison for electron density is reproduced in Fig. 3. The comparison in general is within a factor of 2 except at *V*_rf_ = 150 V and *p* = 133 Pa (1.0 Torr) where the difference seems to be about a factor of 3. In some cases, the reported electron densities also seem to be in reasonable agreement with the values from Kushner and Sommerer [2] even though the trends appear somewhat different. Riley et al. also compared He metastable densities with measurements and found that though the predicted trends (variation with rf voltage) are as seen in experiments, the densities are higher than experimental data by less than a factor of 2 for the triplets and about a factor of 3 for the singlets. The authors suggest this discrepancy is due to the lack of detailed treatment of the high Rydberg levels in their model of the He excitations.

### 3.2 Fluid Model Results

The studies in Refs. [3–5] demonstrated the capability of fluid models to capture key rf discharge phenomena. These one-dimensional models used GEC cell dimensions (electrode gap of 2.54 cm) and computed discharge behavior as a function of pressure and applied rf voltage. The discharge was assumed symmetric and all radial variations were ignored. No comparison against GEC cell measurements were attempted in these early models; for this reason, only a brief summary highlighting key achievements of each work is presented here. Lymberopoulos and Economou [3] were the first to include a detailed balance equation for metastables in fluid simulations of rf discharges. At a pressure of 133 Pa, their results showed that the electron density with metastables included in the model is about six times higher than that without metastables. Stepwise-ionization was shown to be the dominant mechanism for electron production at 133 Pa compared to direct ionization of the ground state atoms. Their results emphasized that neutral transport and reactions must be considered in a self-consistent manner, even in inert gas discharges, in order to capture the observed behavior of rf discharges. Lymberopoulos and Economou also effectively demonstrated an approach based on the zero-dimensional solution of the Boltzmann equation to generate the rate constants needed in fluid models. This approach is more realistic than the Arrhenius expressions based on Maxwellian EEDF commonly used in fluid models until that time. Young and Wu [5] showed that the three-moment fluid model is much more capable of capturing non-equilibrium effects than one- and two-moment models. Comparing their results against Monte-Carlo simulations, they concluded that the three-moment fluid model can predict, within reasonable accuracy, the transition between the *α* and *γ* regimes. Meyyappan and Govindan [4] showed that with rate constants obtained from a zero-dimensional solution of the Boltzmann equation, their three-moment model reproduces excitation wave forms as seen in experiments. They also showed that the three-moment fluid model can reproduce several critical discharge features, previously reported only by PIC simulations, such as mechanisms of electron heating and relation between electron power deposition and ionization. The simulations in Ref. [4] provided some scaling laws for the discharge behavior. Electron density in electropositive discharges scales nearly linearly with rf voltage. Both the rf and ion current also scale linearly with rf voltage. Power deposited to the electrons varies linearly with rf voltage while ion power varies linearly with the square of the rf voltage.

**Fig. 4:**
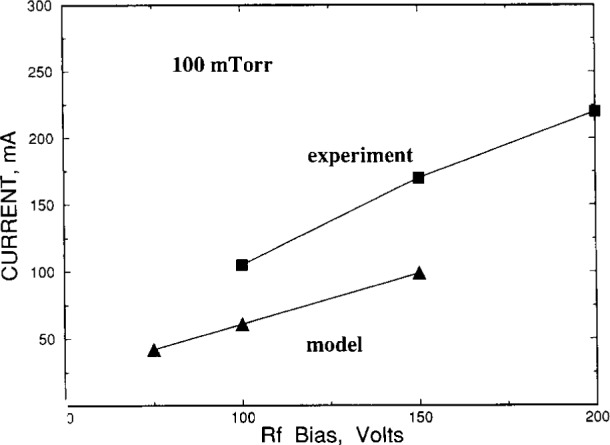
Current-voltage characteristics at 13.3 Pa predicted by a three-moment fluid model. Measurements from Ref. [10] for a symmetric discharge are also shown.

**Fig. 5:**
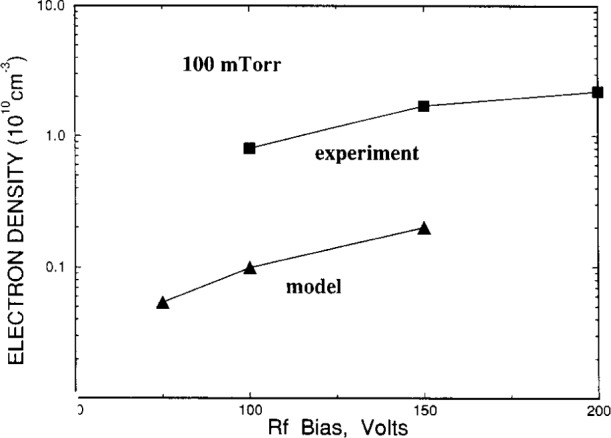
Peak electron density from a three-moment fluid model at 13.3 Pa. Microwave interferometry measurements from Ref. [10] for a symmetric discharge are also shown.

In the remainder of this section, we discuss unpublished results from our research on GEC reference reactor modeling. These results are for a 13.3 Pa (100 mTorr) argon discharge at three zero-to-peak (*V*_rf_) voltages of 75 V, 100 V and 150 V. The results are compared with the current-voltage, microwave interferometry, and optical emission measurements reported in Refs. [10–13]. It is noted that Greenberg and Hebner [10] powered both electrodes in a push-pull fashion and their discharge was fairly symmetric while other works in Refs. [11–13] reported an asymmetric discharge. Since 1-D models are naturally symmetric, we first compare the results of our 1-D model with the results of Greenberg and Hebner [10] in Figs. 4 and 5. Figure 4 shows the zero-to-peak (*I*_rf_) current at 13.3 Pa from our simulations and Ref. [10]. An area of 81 cm^2^ was used to convert the peak current density to peak current [6,10]. The comparison between simulation and measurement is reasonable and well within a factor of two. Figure 5 shows the peak electron density from the simulation and microwave interferometry results reported by Greenberg and Hebner. The line averaged density was converted in Ref. [10] by assuming a uniform electron density confined within the 10 cm of the electrodes. Here, the model results are nearly eight times smaller than the measurements. We notice that at low pressures, there is a significant difference between microwave interferometry and Langmuir probe results. For example, such a comparison by Overzet and Hopkins [11] shows that at 13.3 Pa and an applied peak-to-peak voltage of 230 V, the interferometry result is higher than Langmuir probe density by a factor of 2.5. It is possible that the plasma may not be confined within the electrodes at 13.3 Pa but this alone cannot explain the deviation. The model does not account for secondary electron emission, which may explain the underprediction of density to some extent. In any case, further simulation work is needed for a range of pressures and voltages.

Next we discuss 1-D modeling results for an asymmetric discharge. The governing equations for a 1-D discharge have been extended to include variation of the area of the discharge in asymmetric discharges due to unequal electrode areas. In the case of the GEC cell, the walls of the cell are grounded and effectively increase the area of the grounded electrode. This effect was included in the present study by (a) increasing the area of the grounded electrode and by (b) assuming the area of the discharge varies linearly from the area of the powered electrode to the effective area of the grounded electrode. The area of grounded surfaces in the GEC cell is approximately 34 times the area of the powered electrode. From available experimental data, clearly this is too large an area to be included as is in the effective area of the grounded electrode. All elements of the grounded surface do not contribute equally to increasing the effective area of the grounded mode electrode. In the present study the area of elements of the grounded surface were weighted inversely by the minimum distance from the element to the powered electrode. This choice of weights for the elements of the grounded surface was based on the assumption that the influence of the elemental area of the grounded surface is directly proportional to the mean electric field between the element and the powered electrode and this mean electric field varies inversely with the distance from the powered electrode. Thus,

$$A_{\text{eff}}=\int \left(\frac{d}{l}\right)dA$$

where *d* is the electrode gap, *l* is the minimum distance between the elemental area and the powered electrode and the integration is carried out over all grounded surfaces. An effective area of approximately six times the electrode area was obtained for the GEC cell. Areas of ports in the cell were excluded from the estimate of the effective area of the grounded electrode.

**Fig. 6:**
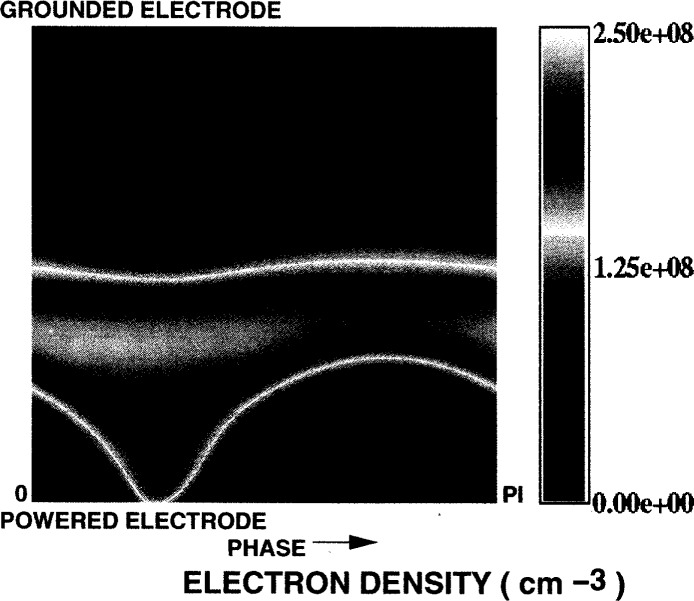
Phase plot of electron density in an argon discharge from a three-moment fluid model. *p* = 13.3 Pa, *V*_rf_ = 100 V.

**Fig. 7:**
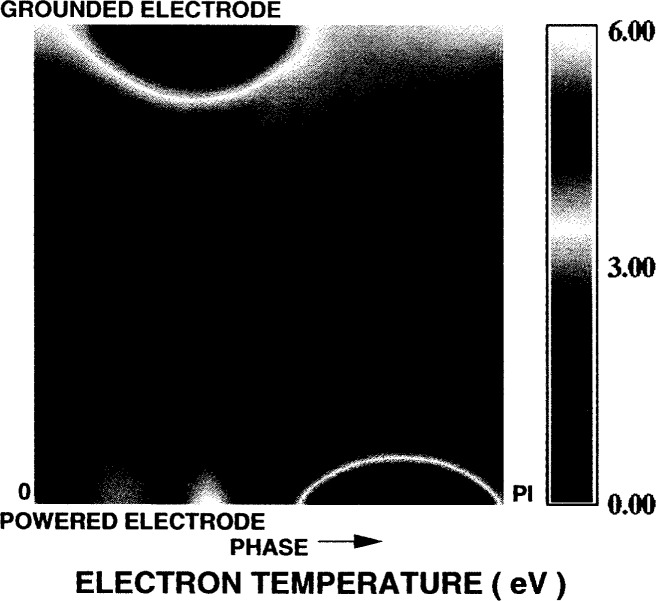
Phase plot of electron temperature in an argon discharge from a three-moment fluid model *p* = 13.3 Pa, *V*_rf_ = 100 V.

**Fig. 8:**
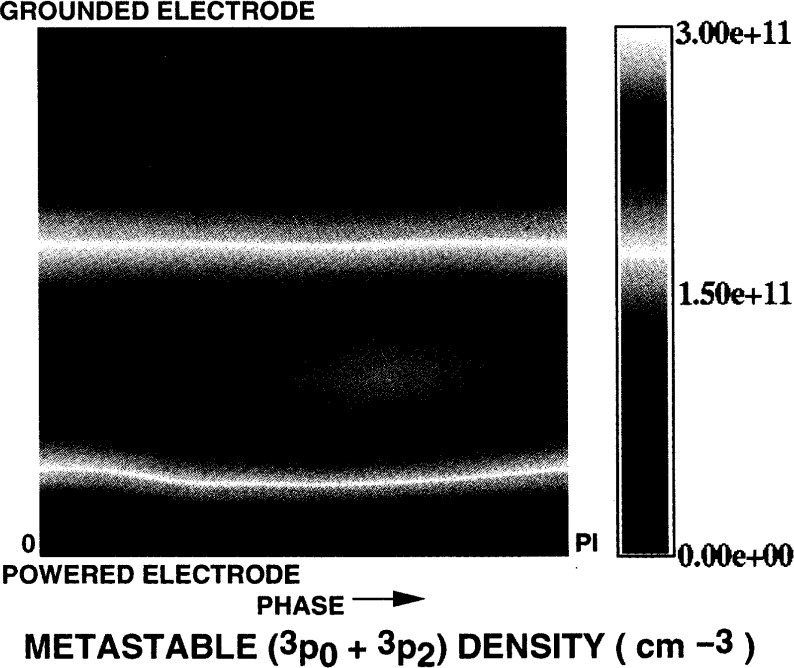
Phase plot of metastable density in an argon discharge from a three-moment fluid model. *p* = 13.3 Pa, *V*_rf_ = 100 V.

**Fig. 9:**
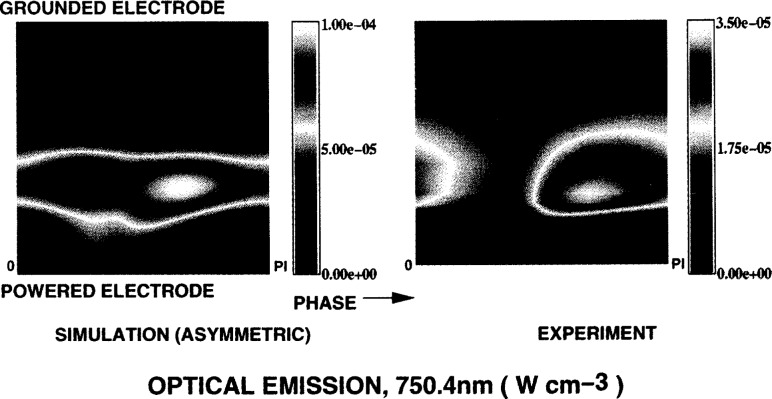
Phase plot of optical emission, argon 750.4 nm line; *p* = 13.3 Pa, *V*_rf_ = 100 V; model (left) and the experimental data from Ref. [14] (right).

Figure 6 shows the phase profile of electron density at 13.3 Pa and *V*_rf_ = 100 V. We notice that the peak occurs off-center toward the powered electrode, as seen in Ref. [12]. In an asymmetric discharge, the densities near the grounded electrode are low throughout the cycle. There is a strong modulation of density near the powered electrode. The peak value predicted here is nearly an order of magnitude different from the measurements in Refs. [11, 12], as discussed earlier. Our model has been bench-marked against simulation results from kinetic schemes and measurements from Godyak for a helium discharge in a workshop organized at the 1993 Gaseous Electronics Conference [13]. Further work at various pressures and bias levels is needed to draw conclusions. Figure 7 shows the corresponding electron temperature profile which is also asymmetric. The bulk of the discharge exhibits an electron temperature of ~4 eV. The metastable (3*p*_0_ + 3*p*_1_) density is plotted in Fig. 8. The peak value is approximately 3×10^17^ m^−3^. The metastable density does not show significant modulation in time. Finally, optical emission due to the 750.4 nm argon line is shown in Fig. 9. The experimental data from Ref. [14] is also shown for comparison. In modeling this process, a number density balance for the 2*p*_1_ excited state was included. Only excitation from the ground state was included using rate constants given by Ferreira et al. [15]; production of 2*p*_1_ excited states through intermultiplet transitions is smaller by an order of magnitude. The Einstein coefficient for the emission process (2*p*_1_ → 1*s*_2_) was taken to be 0.445×10^8^ s^−1^. We note from Fig. 9 that the model predicts the spatial location of the peak very well, indicating that our effective area approach is reasonable. Conventional, symmetric 1-D models would have predicted two spatial peaks displaced symmetrically about the center of the discharge, one at *t* = 0.25 and another at *t* = 0.75 [3, 4]. The model prediction of emission intensity is within a factor of 2–3 of the experimental data. The only deviation is a band of somewhat stronger emission at around *t* = 0.25 when the voltage is at its peak. In the experiment, a dc bias is present across the electrodes, which would tend to decrease the emission at *t* = 0.25. This bias was not included in the simulations.

## 4. Summary

We have presented a review of one-dimensional models of the GEC reference plasma reactor based on a small number of publications in the literature. Most of the studies reviewed were limited to qualitative comparison with available data and the models reproduce many of the observed features. Quantitative comparison between model predictions and measurements has been done in a few cases in terms of current-voltage and line-averaged electron density; here the agreement ranges from reasonable to within an order of magnitude. The 1-D model has also been modified to account for the asymmetry of the GEC cell using an effective area approach which seems to predict the spatial asymmetry of emission profiles reasonably. Modeling techniques, both fluid and kinetics schemes, have improved vastly in the past 5 years. Based on the number of papers at the 1994 Gaseous Electronics Conference reporting on diagnostics in the GEC cell and the preceding articles in this Special Issue, it appears that much more experimental data for a number of gases will be available for model validation. This provides an ideal opportunity for the modelers to fine tune their discharge physics modules and provide a basis for integration with reactor models. There are several challenges ahead in GEC cell modeling: modeling electronegative discharges with proper accounting of various inelastic processes, coupling plasma transport equations with external circuit boundary conditions, investigation of the sensitivity of model results to the boundary conditions, and efficient two-dimensional time-dependent models that would allow routine simulation of GEC and commercial reactors.

### Note added in proof

The predicted electron density values (Figs. 5 and 6) from the three-moment fluid model increase by a factor of two if the ion mobility variation with the electric field is included in the model. The model results then are within a factor of two of the microwave interferometry measurements at pressures of 33.3 Pa and above. At 13.3 Pa, the model predictions now are smaller than the experimental results [10] by a factor of four. However, microwave interferometry measurements at 13.3 Pa in argon from various groups exhibit a considerable disagreement, as reported by Overzet in this Special Issue.
